# DLC1 as Druggable Target for Specific Subsets of Gastric Cancer: An RNA-seq-Based Study

**DOI:** 10.3390/medicina59030514

**Published:** 2023-03-06

**Authors:** Lianlei Yang, Adil Manzoor Bhat, Sahar Qazi, Khalid Raza

**Affiliations:** 1 Department of Gastroenterology, The First People’s Hospital of Linping District, Hangzhou 311100, China; 2 Department of Computer Science, Jamia Millia Islamia, New Delhi 110025, India

**Keywords:** adenocarcinoma, biomarkers, differentially expressed genes, pathways, macromolecular energy landscape, NGS analysis

## Abstract

*Background:* Gastric cancer has been ranked the third leading cause of cancer death worldwide. Its detection at the early stage is difficult because patients mostly experience vague and non-specific symptoms in the early stages. *Methods*: The RNA-seq datasets of both gastric cancer and normal samples were considered and processed. The obtained differentially expressed genes were then subjected to functional enrichment analysis and pathway analysis. An implicit atomistic molecular dynamics simulation was executed on the selected protein receptor for 50 ns. The electrostatics, surface potential, radius of gyration, and macromolecular energy frustration landscape were computed. *Results*: We obtained a large number of DEGs; most of them were down-regulated, while few were up-regulated. A DAVID analysis showed that most of the genes were prominent in the KEGG and Reactome pathways. The most prominent GAD disease classes were cancer, metabolic, chemdependency, and infection. After an implicit atomistic molecular dynamics simulation, we observed that DLC1 is electrostatically optimized, stable, and has a reliable energy frustration landscape, with only a few maximum energy frustrations in the loop regions. It has a good functional and binding affinity mechanism. *Conclusions*: Our study revealed that DLC1 could be used as a potential druggable target for specific subsets of gastric cancer.

## 1. Introduction

Gastric cancer begins in the mucus-producing cells on the inside lining of the stomach. Stomach adenocarcinoma is the leading cause of cancer death across the globe [1]. According to GLOBOCAN 2020, gastric cancer is ranked the fifth most occurring cancer (5.6% of all cancers) after breast, lung, prostate, and colon cancer [2]. It is the third most death-causing cancer in both males and females, yielding 7.7% of all cancer-related deaths [2]. Globally, its case–fatality ratio is higher than common malignancies such as colon, breast, and prostate cancers. It has been seen that the prevalence of gastric cancer is increasingly frequent in males, as compared to females, i.e., 15.8 per million in males, while 7.0 per million in females [2]. Gastric cancer is a highly heterogeneous disorder, which usually arises from the epithelium of the stomach and is mostly found near the cardia part of the stomach [3]. Gastric cancer has been classified into various subtypes by the World Health Organization (WHO), which include mucinous adenocarcinoma, tubular adenocarcinoma, poorly cohesive carcinoma, and mixed carcinoma [4]. The basis for this classification of subtypes is histological motifs. There are two variants of gastric cancer based on pathology, such as intestinal and diffuse [3]. There are various causes of gastric cancer, ranging from environmental risk factors to hereditary factors [5]. Various risk factors have been found to be associated with the occurrence of gastric cancer, such as dietary habits, the consumption of liquor, red meat, and smoking [6]. Infection by Helicobacter pylori and the Epstein–Barr virus are also included in the risk factors for gastric cancer. The process of gastric cancer development takes place in several steps, ranging from atrophic gastritis to dysplasia, adenoma, and, finally, adenocarcinoma [7,8]. The progression of gastric cancer occurs due to abnormalities in the protein-encoding genes that control and regulate the normal growth of gastric cells [9]. According to The Cancer Genome Atlas (TCGA), there are four molecular subtypes of gastric cancer, namely the Epstein–Barr virus, microsatellite instability, genomically stable, and chromosomal instability. In the early stages, the tumor cells are restricted to the superficial layer of the stomach, and most patients are asymptomatic. Despite advances in diagnosis, gastric cancer is more often detected when it invades the smooth muscle layer [10]. The majority of patients suffer from more indistinct and general symptoms in their early stages. In the advanced stages, symptoms such as loss of weight, abdominal pain, loss of appetite, stomach fullness, dyspepsia, anemia, and denial of meat-based foodstuffs are seen [11]. Gastric cancer is not usually detected and diagnosed in the early stages, although there are several diagnostic methods available, such as a physical examination, radiological diagnosis, genetic screening, and endoscopic diagnosis [9]. During diagnosis, the depth of tumor infiltration is the most significant factor for its prognosis. To tackle the menace of gastric cancer, various therapeutic procedures have been developed from time to time, and currently, the various therapies for the treatment of gastric cancer include surgical therapy, radiotherapy, endoscopic therapy, neoadjuvant chemotherapy, and immunotherapy [12]. Most of these therapies are not effective due to the meager prognosis and low survival rate of gastric cancer patients; thus, a new look at the results of RNA-seq data analysis, along with pathway and functional enrichment analysis involving tools and techniques of bioinformatics, can play an indispensable role in establishing methodologies to explore the root cause of gastric cancer at the molecular level and to attain fundamental anticipation [6,13]. The RNA-seq from next-generation sequencing (NGS) facilitates the discovery of potential druggable targets in various diseases, including cancers, that can be further validated in the experimental setup for their efficacy. Due to the lack of potential druggable targets in gastric cancer, there is a need to explore RNA-seq for its discovery.

In this paper, we carried out an in-depth analysis of gastric cancer by deploying transcriptomic (RNA-seq data) analysis tools to explore potent biomarkers that are associated with gastric cancer. The differentially expressed genes (DEGs) obtained in this RNA-seq data analysis pipeline were then subjected to the DAVID functional enrichment analysis to identify the disease pathways and molecular processes in which identified DEGs are involved to establish potential biomarkers of gastric cancer. Further, the identified potential biomarkers were subjected to a stability and binding affinity study using a molecular dynamics simulation.

## 2. Materials and Method

### 2.1. Datasets

A dataset of 20 samples was considered, including 10 gastric cancer samples and 10 normal gastric tissue samples. RNA-seq data for gastric cancer were obtained from the Sequence Read Archive (SRA) repository of the NCBI. Table 1 presents RNA-seq datasets of two experimental conditions (Tumor vs. Normal), along with their Accession No. and the total number of bases.

### 2.2. Data Processing Pipeline for DEGs’ Identification

**Figure 1:**
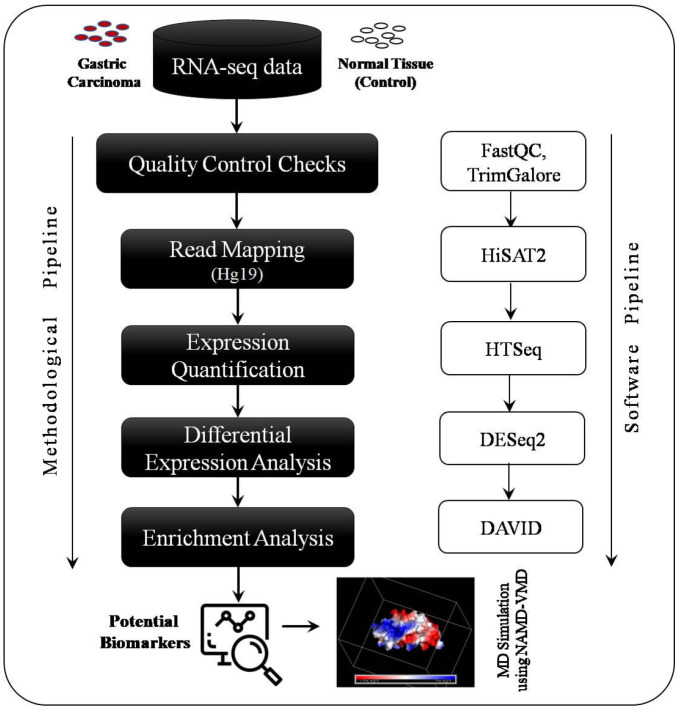
RNA-seq analysis methodological and software pipeline.

The complete methodological and software pipeline used in this study is shown in Figure 1 and explained as follows:

Quality control checks: The quality control (QC) checks were carried out using a FASTQC software tool (https://www.bioinformatics.babraham.ac.uk/projects/fastqc/,accessed on 15 July 2022) for each sample. All the samples passed the QC checks, as per recommended Phred quality scores (q-score ≥ 30). In one of the samples, the adaptor content was not in the desired range; therefore, the quality of this sample dataset was improved using the Trim Galore tool (https://www.bioinformatics.babraham.ac.uk/projects/trim_galore/, accessed on 15 July 2022), and then the quality was rechecked using the FASTQC tool, and it passed the check.

Read Mapping to Hg19 genome: After the QC, the sample reads were mapped to the human genome Hg19 using a HiSAT2 read mapper [14]. HiSAT2 is a sensitive and fast-read mapping software used for both DNA and RNA sequence reads, based on an extension of the Burrows–Wheeler Transformation (BWT) for graphs [15]. HiSAT2 uses a graph FM (GFM) index and a large set of small GFM indexes in order to cover the whole genome [15].

Read Count Quantification: After mapping the reads to the reference genome, we carried out a read count quantification using an HT-SEQ tool [16] using aligned reads obtained in the mapping step. The output was a read count file which was then subjected to normalization using DESeq2 [17].

Differential Expression Analysis*:* We utilized one of the most popular and widely used tools for differential expression analysis called DESeq2 [17]. The adjusted *p*-value was obtained by the Benjamini and Hochberg method to control the probability of false positives, and the log of fold-change (logFC) was utilized to detect differentially expressed genes (DEGs). Refer to [18] for a detailed discussion on the logFC and *p*-value.

### 2.3. GO and KEGG Pathway Enrichment Analysis

The identified DEGs were subjected to Gene Ontology (GO) and KEGG pathway enrichment analysis using a DAVID tool (https://david.ncifcrf.gov/, accessed on 15 July 2022). DAVID [19] is a GO enrichment analysis tool providing a comprehensive annotation of biological functions for both genes and proteins and includes a biological process (BP), cellular component (CC), and molecular function (MF). GO enrichment analysis answers which GO terms are overrepresented or underrepresented based on pre-defined and pre-stored annotation information in the database [18].

### 2.4. Molecular Dynamics Simulation

To check for overall stability, electrostatics, and the macromolecular frustration landscape, we executed an implicit atomistic molecular dynamics simulation using QwikMD, a plugin available in NAMD-VMD software [20]. We deployed an implicit solvent-based atomistic molecular dynamics mainly for two reasons—(a) it is computationally faster than explicit solvent simulations, and (b) it provides a rapid and enhanced conformational sampling, viz. because of generalized Born approximations [21]. Since DLC1 was selected for simulation, the tertiary structure was retrieved from RCSB PDB (PDB Id: 2DKY) (https://www.rcsb.org/structure/2DKY, accessed on 15 July 2022). It is a solution NMR structure of the SAM-domain of the Rho-GTPase-activating protein 7 (homo sapiens), which has a single chain A with a total of 91 residues.

The simulation was executed at a salt (NaCl) concentration of 0.15 mol/L under an implicit solvent system. The simulation was initiated with an equilibration from 60 K to 300 K for 10 ns, and the production simulation was recorded from 40 ns at 300 K. All the simulation results were saved periodically at every 2fs step. The production simulation trajectory files were used for electrostatic and root-mean-square fluctuation (RMSF) analyses using VMD1.9.3 (https://www.ks.uiuc.edu/Research/vmd/vmd-1.9.3/, accessed on 15 July 2022). The molecular mechanics generalized Born surface area (MM-GBSA) approach was computed over the simulation time using a PyMol plugin, the adaptive Poisson–Boltzmann solver (APBS) (https://pymolwiki.org/index.php/APBS_Electrostatics_Plugin, accessed on 15 July 2022). The radius of gyration (RoG) was calculated separately using the SCFbio ROG webserver (http://www.scfbio-iitd.res.in/software/proteomics/rg.jsp, accessed on 15 July 2022). For macromolecular energy frustration landscape analysis, we used an online web server named the Frustratometer server [22].

## 3. Results

To predict the potential biomarkers for gastric carcinoma, we analyzed RNA-seq data from next-generation sequencing (NGS) taken from the NCBI-SRA database. We considered 10 samples from gastric carcinoma and 10 samples from the normal control. After the necessary QC and read quality improvement, the complete data processing pipeline, mentioned in Figure 1, was executed. The results of each step are described in the following section.

### 3.1. Read Mapping

After the mapping of the sample reads to Hg19, it was observed that there was approximately a 95% overall alignment rate. Initially, 99.82% of the reads aligned discordantly, but when the parameters were set again, the overall alignment rate improved drastically.

### 3.2. Identification of Differentially Expressed Genes

**Figure 2:**
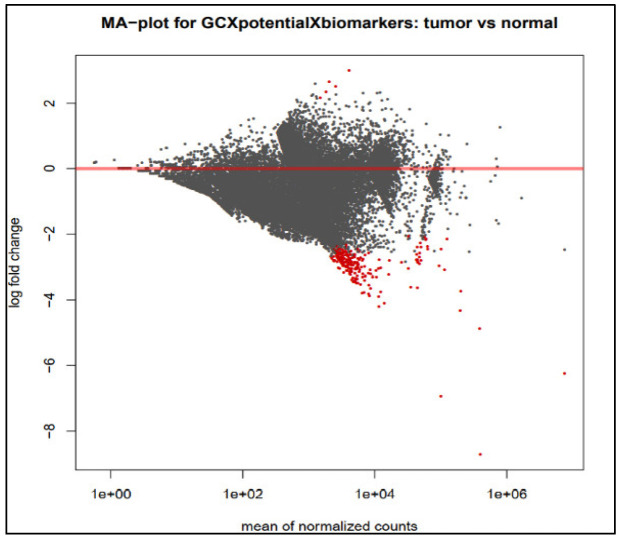
MA plot of differential expression analysis showing the log_2_FC over the mean of normalized read counts. The red dots display highly significant DEGs.

**Figure 3:**
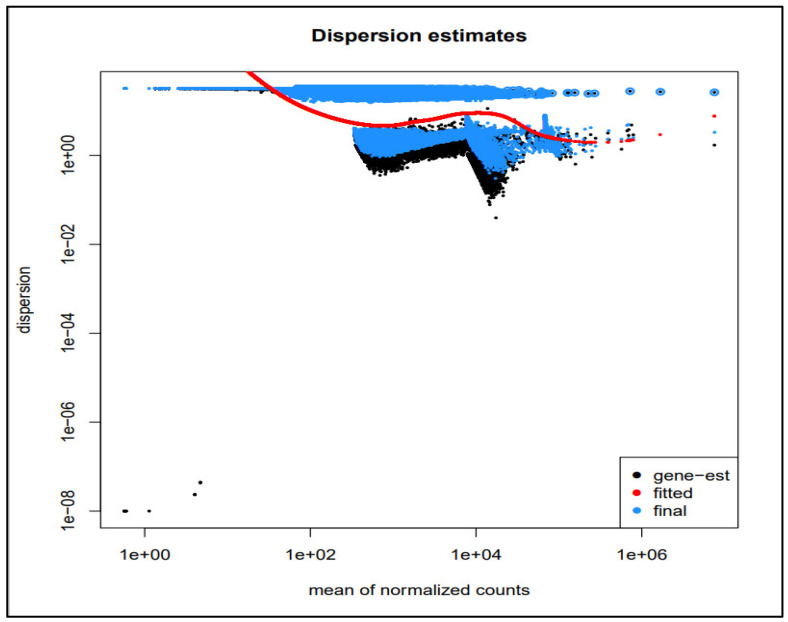
Dispersion plot for the mean of normalized read counts.

After the read mapping, read count quantification, and its normalization, differential expression analysis was performed using the DESeq2 tool. The MA plot of the DEGs has been depicted in Figure 2, where the red color shows the significant DEGs. Figure 3 depicts the dispersion plot of the mean normalized read counts. To shortlist the most significant DEGs over the two sample types, we applied a threshold of −2 ≥ logFC ≥ 2 and *p*-value ≤ 0.05. As a result, we obtained 569 DEGs; most of them are down-regulated, while few are up-regulated genes. Out of these DEGs, the top 100 genes were selected for further downstream analysis.

### 3.3. GO and KEGG Pathway Enrichment Analysis

To understand the biological function and the pathways of the identified DEGs, we performed the GO function and KEGG enrichment analysis. Further, Gene Set Enrichment Analysis (GSEA) was performed on the gene lists. The GO results showed that the DEGs were significantly enriched in the cytoplasm, cell–cell junctions (CC), DNA replication protein phosphorylation, metabolic processes, cation transmembrane transport (BP), and the protein binding, ATP-binding, and RNA polymerase ii core promoter proximal region sequence-specific DNA binding (MF) (Table 2 and Table 3). The KEGG pathway enrichment analysis showed that mostly the identified DEGs were prominent in the KEGG and Reactome pathways. The most prominent GAD disease classes were cancer, metabolic, chemdependency, and infection. The results are presented in Table 2, Table 3 and Table 4.

Since the up-regulation or down-regulation of genes is associated with the initiation and progression of diseases such as in our gastric cancer, as a result of the RNA-seq data analysis, genes with varying levels of expression were detected in the form of DEGs. The DEGs obtained in our study were filtered using the logFC and *p*-value parameters to identify the prominent genes. We found almost 20 genes whose expression levels were increased in the gastric cancer samples significantly, and we also identified the genes whose expression levels decreased significantly and play a pivotal role in various cases of gastric cancer. When the functional and gene enrichment analyses were carried out on the list of the top up-regulated and down-regulated genes, it was found that most of the genes were clustered in the cancer, metabolic, chemdependency, and infection GAD disease classes. One of the genes that were highly down-regulated in our study was DLC1 (deleted in liver cancer 1), showing more than an 8-fold change and a *p*-value ≤ 0.0001, and it has been verified from the literature search that a low expression of DLC1 is associated with gastric cancer [23,24]. The low expression of DLC1 signifies an advanced tumor-node-metastasis stage, node metastasis, increased tumor size, increased lymph deeper tumor invasion, and an elevated distant metastasis rate [23]. According to the Human Protein Atlas [25], DLC1 is a cancer-related tumor suppressor protein that encodes a GTPase-activating protein (GAP). Its function has been annotated as the GAP for the small GTPases RHOA, RHOB, RHOC, and CDC42, terminating their downstream signaling that plays a critical role in several biological processes, including cell migration and proliferation. Further, DLC1 functions as an activator of the phospholipase PLCD1 [25]. Hence, apart from several other genes, we report DLC1 as a key regulator that can act as a potential biomarker for the diagnosis and therapeutics of gastric carcinoma.

### 3.4. Stability Study of DLC1

**Figure 4:**
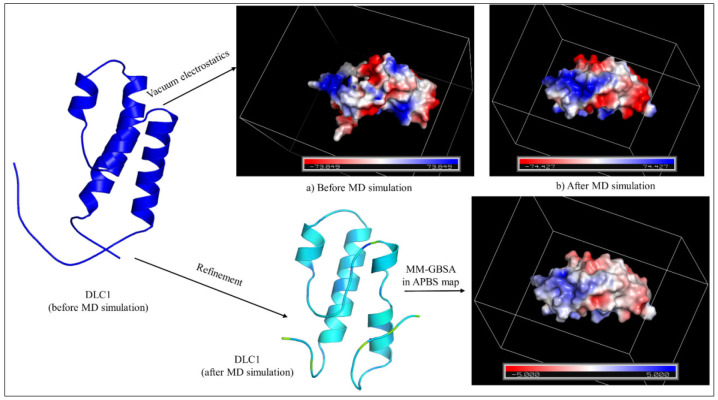
Vacuum electrostatic and MMGBSA potential of DLC1: (**a**) vacuum electrostatics before MD simulation and (**b**) vacuum electrostatics after MD simulation. The light blue-colored tertiary structure represents the refined structure of DLC1 after the MD simulation, along with the MMGBSA electrostatic potential in an APBS map.

To evaluate the overall stability of DLC1, we executed a successful atomistic molecular dynamics simulation for 50 ns. To decide the overall stability of a structure, we often rely on interactions, such as hydrogen bonding, hydrophobic interactions, salt bridges, and pi-interactions; however, the electrostatic and surface potential are often neglected. Electrostatics and surface potential computations play a massive role in understanding the overall configuration of a macromolecule; they can also describe the folding and unfolding of protein structures. Change in the pH also dictates the energy landscape and fluctuations observed in a simulated structure. In our case, DLC1, after refinement, seems to have gained good stability and has minimum steric clashes and hindrances. The vacuum electrostatics of the DLC1 (PDB Id: 2KDY) after refinement gained a greater negative charge (−74.427 to +74.427), which was recorded as −73.849 to +73.849 before the molecular dynamics (MD) simulation. The electrostatic potential was recorded to lie between −599.566 to 528.405. Figure 4 displays the surface potential and the MM-GBSA electrostatic potential in the form of the APBS maps below. Table 5 summarizes the detailed results of the DLC1 post-MD simulation.

**Figure 5:**
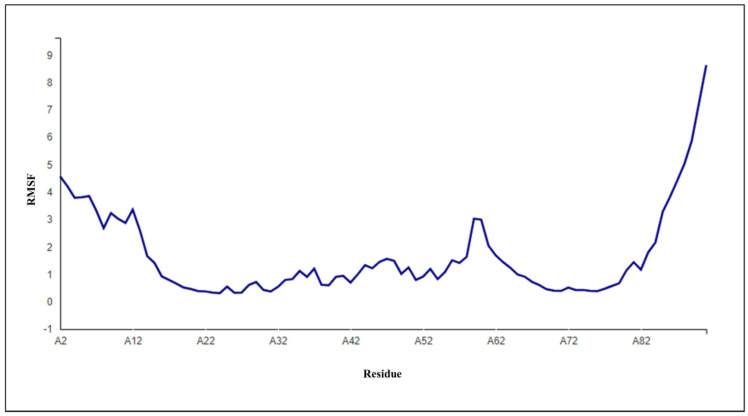
Root-mean-square fluctuation (RSMF) plot of the simulated DLC1 structure.

One of the essential parameters in assessing the stability is by computing the root-mean-square deviation (RMSD) as it defines the hinges and peaks that are present in the structure during the simulation analysis. The RMSD analyses help us to understand how well the structure has been equilibrated before the final production in a molecular dynamics simulation. We observed a few hinges and peaks in the simulated structure of DLC1 (refer to Table 5). The RMSD was recorded for the first 10 ns, which included the loop and major helices of the DLC1 structure. After compiling the essential energies and electrostatics, it was found that the DLC1 had shortened itself by refining the long-stretched loop region, which eventually impacted its overall folding mechanism. Additionally, the total energy reflects good stability and suggests that the DLC1 was electrostatically optimized to function as a reliable binding protein structure. To check whether the refined structure was simulated, we used the accuracy score. Accuracy scores generally lie between 0–1. The refined structure of DLC1 was relatively accurate to the original DLC1 structure. The major changes in the loop regions and short helix region must have caused the accuracy score to be around 0.88 (refer Table 5). Figure 5 below represents the root-mean-square fluctuation (RMSF) plot of the refined DLC1 structure. It is evident that there were only a few hinges and peaks present after the refinement.

**Figure 6:**
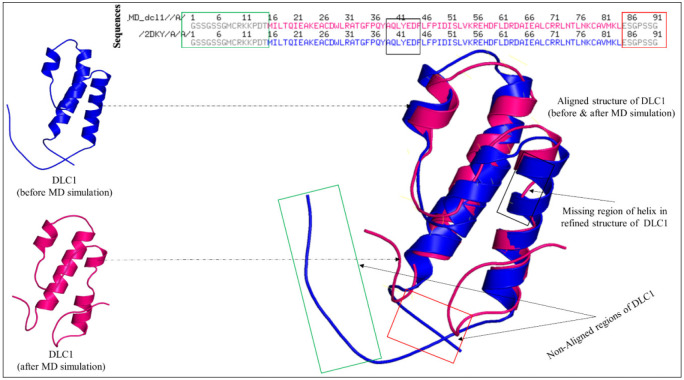
Aligning the before and after molecular dynamics-simulated structures of DLC1. There were a few regions in the refined structure that did not align perfectly with the original DLC1 protein.

To check the refinement status, we aligned both structures before and after the molecular dynamics simulation structures of DLC1. Figure 6 represents the aligned structure with the non-aligned residual segments. After the alignment, we observed that the DLC1, before the simulation analysis, had longer loops and helices. Some residues that formed the main loop present in the DLC1 (before the simulation), starting from residues 1–15 (GSSGSSGMCRKKPDT), seemed to have been shortened in the refined structure, as this region did not align with the refined structure of the DLC1. Another loop region, starting from residues 85–91 (ESGPSSG), tended to have folded on the upper hand, which was different from the original DLC1 (refer to Figure 6). Also, a short region helix, starting from residues 39–44 (AQLYEDF), turned into a loop in the refined structure, unlike a complete helix, as observed in the original DLC1 protein structure. It is evident that the folding of the loops changed as refinement. This could be possible because of the electrostatic potential that has been known to enhance the protein folding and binding potential of proteins [26].

**Figure 7:**
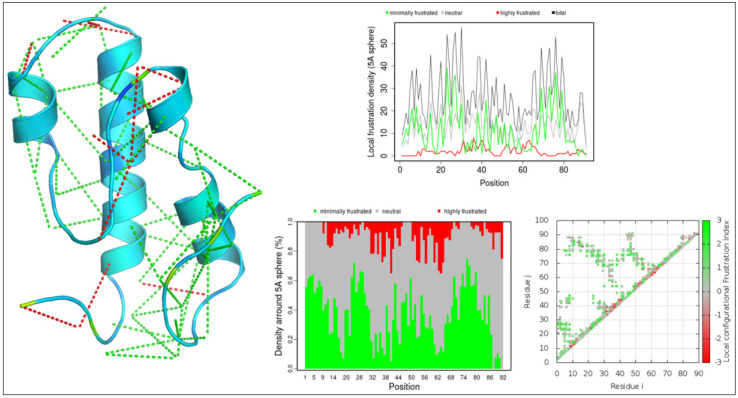
Energy frustration landscape in DLC1. It was observed that there were only a few energy frustrations present in the refined DLC1 protein structure. Maximum energy frustrations are represented in red, while minimum energy frustrations are shown in green. The grey color signifies a neutral landscape.

The macromolecular energy frustration landscape is a phenomenon mainly observed in post-molecular dynamics simulations. Changes in the energy landscape define the simulation procedure and how well the structure has been refined. It is mainly studied to understand and gain insight into the function, binding affinities, and overall behavior of the macromolecules. Minimum energy frustration defines a stable and reliable binding of the protein, while maximum energy frustration refers to a low binding affinity and poor functional regions present in a protein [27]. DLC1 showcased an overall good energy landscape as it contained only a few maximum energy frustration links (mainly in the loops). Figure 7 depicts the overall energy landscape of DLC1. The maximum energy frustrations are represented in red, while the minimum energy frustrations are shown in green. The local energy frustration was mainly observed in the initial loopy regions. Overall, the simulated and refined structure of DLC1 showcased a good energy landscape and proved to have a reliable functional dynamic.

## 4. Discussion

In this RNA-seq-based study, we obtained a number of differentially expressed genes (DEGs) with varying levels of expression. We found almost 20 up-regulated genes and a number of down-regulated genes, playing a pivotal role in various cases of gastric cancer. The functional and gene ontology (GO) enrichment analysis suggests that most of the genes are clustered in the cancer, metabolic, chemdependency, and infection GAD-disease classes. The abnormalities in the metabolic pathways are associated with the initiation and progression of some diseases, such as cancer, which validates our GAD disease class (metabolic) in our study. There are a number of prominent genes found in our study that are associated with adenocarcinoma (GC). The GO analysis suggests that a number of DEGs are associated with neurological mechanisms and brain metabolic pathways. This is due to the fact that most deadly diseases of brain-like encephalitis and meningitis are caused by the Epstein–Barr virus, and the same virus was found to be involved in various sub-types of gastric cancer by the TCGA project [28], the result of which is the convergence of gastric cancer and brain pathways at various stages of the functional analysis. The highly dysregulated gene predicted in gastric cancer is DLC1, having more than an 8-fold change in its expression (*p*-value ≤ 0.0001) and being a down-regulated gene, and a low expression of DLC1 is associated with gastric cancer [24]. The low expression of DLC1 signifies an advanced tumor-node-metastasis stage, node metastasis, increased tumor size, increased lymph deeper tumor invasion, and an elevated distant metastasis rate [23]. In one of the studies carried out by YuqiSu and collaborators [23], DLC1 was identified to be significantly lower in the gastric cancer samples, mainly in tumors of higher disease stages, with increased invasion, distant metastasis, and lymph node metastasis. Further, DLC1-negative gastric cancer patients exhibit shorter survival rates and higher recurrence risks, thereby indicating that a low expression of the DLC1 gene is associated with gastric cancer [23]. This indicates that DLC1 can be used as a potential druggable target for specific subsets of gastric cancer, as reported in [24]. In a study by Kim et al. [29], it is reported that seven out of nine gastric cancer cell lines do not express the DLC1 mRNA, but it does contain the DLC1 gene. The methylation study suggests that five out of the seven DLC1 non-expressing gastric cancer cell lines were methylated in the DLC1 CpG island, which is not uncommon in gastric cancer [29].

Helicobacter infection may lead to chronic gastric inflammation; therefore, it may increase the risk factor for the development of gastric carcinoma. The role of DLC1 in Helicobacter-related gastric cancer was studied in [30], where DLC1 gt/+ mice showed increased gastric inflammatory infiltration. Further, the study demonstrated that DLC1 is transcriptionally down-regulated by CagA, which promotes oncogenic effects and constitutes DLC1 as an early molecular marker for Helicobacter-related gastric disease. Furthermore, the suppression of tumor growth using an inhibitor of the RHOA downstream effector, ROCK, suggests DLC1 as a druggable target for gastric cancer [30].

Several studies suggest the vital role of autophagy in cancers, including gastric cancer. A differential expression analysis of autophagy-related genes (ARGs) in gastric cancer was carried out by Li and collaborators [31], who report a total of seven prognosis-related ARGs, including DLC1, as independent prognostic markers for gastric cancer [31]. A biomarker-based retrospective study [32] provides support for the use of the lymphocyte-to-monocyte ratio (LMR) as a novel predictor for colorectal liver cancer (CLC) treated with radiofrequency ablation (RFA). A patient shows better survival outcomes after RFA for a baseline LMR > 3.96% [32].

Electrostatics and surface potential computations play a massive role in understanding the overall configuration of a macromolecule; they can also describe the folding and unfolding of protein structures. A change in the pH also dictates the energy landscape and fluctuations observed in a simulated structure. In our case, DLC1, after the refinement, seems to have gained good stability and has minimum steric clashes and hindrances. After the simulation, we aligned the before and after tertiary structures of DLC1 to compare and deduce the refinement. We observed that some residues that formed the main loop present in DLC1 (before the simulation), starting from residues 1–15 (GSSGSSGMCRKKPDT), seemed to have been shortened in the refined structure, as this region did not align with the refined structure of the DLC1. Another loop region, starting from residues 85–91 (ESGPSSG), tended to have folded on the upper hand, which is different from the original DLC1 (refer to Figure 6). Additionally, a short region helix, starting from residues 39–44 (AQLYEDF), turned into a loop in the refined structure, unlike a complete helix, as observed in the original DLC1 protein structure. The folding of the loop changed with refinement. This could be possible because of the electrostatic potential, which has been known to enhance the protein folding and binding potential of proteins [26]. The macromolecular energy landscape study shows minimum frustrations that define a stable and reliable binding of the protein, while the maximum energy frustration refers to a low binding affinity and poor functional regions present in a protein [27]. The DLC1 showcased a good energy landscape, as it contained only a few maximum energy frustration links. It is obtained that DLC1 as a receptor is electrostatically optimized and stable, with good functional dynamics and binding affinity for small molecules, ligands, and drugs. However, it may be further validated and assessed clinically as to how it can be used as a therapeutic in the treatment of gastric cancer.

## 5. Conclusions

As a result of RNA-seq data analysis, a list of DEGs has been identified, along with a few potential biomarkers and druggable targets for gastric cancer, as identified by functional enrichment analysis using the DAVID analysis tool. The expression of DLC1 was identified to be significantly lower in the gastric cancer samples. DLC1-negative gastric cancer patients exhibit shorter survival rates and higher recurrence risks, thereby indicating that a low expression of the DLC1 gene is associated with gastric cancer. This indicates that DLC1 can be used as a potential druggable target for specific subsets of gastric cancer. The results can be further validated by some PCR techniques in vitro to identify and determine the molecular mechanism of the disease and potential drug targets, which will ultimately enhance the diagnosis and treatment of gastric adenocarcinoma in the initial stages of its development. Further validation of DLC1 and other significant DEGs identified can be conducted, which can provide new research directions for the detection and treatment of cancer and, at the same time, improve the prognosis of gastric cancer using these biomarkers. Further, we also discerned that DLC1 as a receptor is electrostatically optimized and stable, with good functional dynamics having a good binding affinity for small molecules, ligands, and drugs. It can be further studied clinically to assess how it can be used as a therapeutic in the treatment of gastric cancer.

## Figures and Tables

**Table 1:** RNA-seq datasets (gastric cancer and normal tissue samples).

Experiment Accession No.	Sample Type	Total Bases (in Millions)
SRX11368669	Gastric cancer	945.80
SRX11368670	Gastric cancer	1457.514
SRX11639389	Gastric cancer	8610.47
SRX11639390	Gastric cancer	9061.85
SRX11799736	Gastric cancer	898.46
SRX12181742	Gastric cancer	820.53
SRX12181743	Gastric cancer	1004.16
SRX12181745	Gastric cancer	1325.47
SRX6479200	Gastric cancer	2080.26
SRX6479201	Gastric cancer	2386.86
SRX3808892	Normal control	10,655.83
SRX3808894	Normal control	13,557.01
SRX3808896	Normal control	13,036.12
SRX3808898	Normal control	14,177.99
SRX3808900	Normal control	14,125.66
SRX3808902	Normal control	13,982.29
SRX9904379	Normal control	4659.08
SRX9904380	Normal control	3183.77
SRX9904381	Normal control	2834.01
SRX9904382	Normal control	4591.04

**Table 2:** Gene ontology (GO)-based biological process enrichment analysis (*p*-value ≤ 0.05).

Category	Term	Gene Count	%	*p*-Value	Benjamini
GOTERM_BP_DIRECT	Cation Transmembrane Transport	4	2.9	0.0022	1.0
GOTERM_BP_DIRECT	DNA Replication	5	3.6	0.0097	1.0
GOTERM_BP_DIRECT	Regulation of cardiac rate by cardiac conduction	3	2.2	0.0150	1.0
GOTERM_BP_DIRECT	Membrane depolarization during atrial cardiac muscle cell action potential	2	1.4	0.0160	1.0
GOTERM_BP_DIRECT	Regulation of endothelial tube morphogenesis	2	1.4	0.0210	1.0
GOTERM_BP_DIRECT	Positive regulation of cell adhesion	3	2.2	0.0220	1.0
GOTERM_BP_DIRECT	Membrane depolarization during AV node cell action potential	2	1.4	0.0270	1.0
GOTERM_BP_DIRECT	Chromatin-mediated maintenance of transcription	2	1.4	0.0470	1.0
GOTERM_BP_DIRECT	Negative regulation of excitatory post-synaptic potential	2	1.4	0.0470	1.0

**Table 3:** Functional categories analysis (*p*-value ≤ 0.05).

Category	Term	Gene Count	%	*p*-Value	Benjamini
UP_SEQ_FEATURE	Compositionally biased region: Ser-rich	9	6.5	0.0024	1
UP_SEQ_FEATURE	Compositionally biased region: Poly-Ser	9	6.5	0.0056	1
UP_SEQ_FEATURE	Region of interest: HA-stretch	2	1.4	0.0110	1
UP_SEQ_FEATURE	Splice variant	54	39.1	0.0110	1
UP_SEQ_FEATURE	Domain: Leucine-zipper	4	2.9	0.0220	1
UP_SEQ_FEATURE	Nucleotide phosphate-binding region: ATP	11	8	0.0370	1
UP_SEQ_FEATURE	Sequence variant	76	55.1	0.0380	1
UP_SEQ_FEATURE	Compositionally biased region: Gln-rich	4	2.9	0.0470	1

**Table 4:** UP_keywords functional category analysis result (*p*-value ≤ 0.05).

Category	Term	Gene Count	%	*p*-Value	Benjamini
UP_KEYWORDS	Alternative splicing	71	51.4	0.0024	0.4800
UP_KEYWORDS	Transferase	18	13.0	0.0069	0.5100
UP_KEYWORDS	Coiled-coil	26	18.8	0.0120	0.5100
UP_KEYWORDS	ATP-binding	15	10.9	0.0130	0.5100
UP_KEYWORDS	Polymorphism	75	54.3	0.0150	0.5100
UP_KEYWORDS	Cytoskeleton	13	9.4	0.0150	0.5100
UP_KEYWORDS	Nucleotide-binding	17	12.3	0.0220	0.6300
UP_KEYWORDS	Cell junction	9	6.5	0.0250	0.6300
UP_KEYWORDS	Phosphoprotein	54	39.1	0.0290	0.6600
UP_KEYWORDS	Obesity	3	2.2	0.0330	0.6800
UP_KEYWORDS	Brugada syndrome	2	1.4	0.0500	0.9400

**Table 5:** Energy landscape and electrostatic computations calculated after MD simulation for DLC1.

***DLC1***	**Accuracy**	**Generalized Born Self-Energy (kJ)**	**Coulomb Energy (kJ)**	**Electrostatic Solvation Energy (kJ/mol)**	**Solvent Accessible Surface Area (SASA) (A2)**	**Total Energy (kJ/mol)**	**RMSD**	**ROG**
	0.88	−10,212.9680	−24,329.9612	−6586.7651	5535.538	−28,373.0488	0.59 ± 0.34	12.92

## Data Availability

The data are publicly available at NCBI-SRA and can be downloaded from the link: https://www.ncbi.nlm.nih.gov/sra by providing the accession no.
